# Trait expression and signatures of adaptation in response to nitrogen addition in the common wetland plant *Juncus effusus*

**DOI:** 10.1371/journal.pone.0209886

**Published:** 2019-01-04

**Authors:** Jennifer Born, Stefan G. Michalski

**Affiliations:** Department of Community Ecology (BZF), Helmholtz Centre for Environmental Research – UFZ, Halle, Germany; Technical University in Zvolen, SLOVAKIA

## Abstract

Wetland ecosystems are known to mitigate high nutrient loadings and thus can improve water quality and prevent potential biodiversity loss caused by eutrophication. Plant traits affect wetland processes directly through effects on accumulation or metabolization of substances, and indirectly by affecting microbial transformation processes in the soil. Understanding the causes and consequences of intraspecific variation in plant functional traits and associated ecosystem processes can aid applied ecological approaches such as wetland restoration and construction. Here we investigated molecular variation and phenotypic variation in response to three levels of nitrogen availability for a regional set of populations of the common wetland plant *Juncus effusus*. We asked whether trait expression reveals signatures of adaptive differentiation by comparing genetic differentiation in quantitative traits and neutral molecular markers (*Q*_ST_—*F*_ST_ comparisons) and relating trait variation to soil conditions of the plant’s origin. Molecular analyses showed that samples clustered into three very distinct genetic lineages with strong population differentiation within and among lineages. Differentiation for quantitative traits was substantial but did not exceed neutral expectations when compared across treatments or for each treatment and lineage separately. However, variation in trait expression could be explained by local soil environmental conditions of sample origin, e.g. for aboveground carbon-to-nitrogen (C:N) ratios, suggesting adaptive differentiation to contribute to trait expression even at regional level.

## Introduction

Wetland ecosystems are known to mitigate high nutrient loadings and thus can improve water quality and prevent potential biodiversity loss caused by eutrophication [1]. The potential of wetlands to limit environmental damages is more and more recognized also by applied approaches using managed and constructed wetlands [2]. Two processes are primarily driving the reduction of high nutrient loads by wetlands: First, a direct accumulation of substances by the vegetation, and second microbial transformation processes [3, 4]. For example, high inorganic nitrogen loads can be reduced by denitrification processes with anaerobic soil microorganisms converting nitrate to nitrogen gas that is lost to the atmosphere [5]. Plant traits can directly and indirectly affect the soil microbiome and its activity and hence microbial N processing [6]. For example, the quantity and quality of leaf litter as well as root exudates can influence soil microclimate thus affecting soil microorganisms [7, 8]. The effect of plant functional traits and their interaction with the soil microbiome on the reduction of contaminants in wetland ecosystems is well studied with respect to among-species variability, e.g. for the efficiency of nitrogen and phosphorus retention [9, 10], root traits [11] and for the degradability of plant tissue [12, 13]. However, recently an increased number of studies demonstrate that also intraspecific variation in functional traits can be highly variable even at regional and locale scale [14, 15].

Understanding the causes and consequences of this intraspecific variation is not only a fundamental challenge for ecological research but it can also aid applied approaches in wetland restoration and construction because it is increasingly recognized as important driver of ecosystem functioning [16, 17]. Across a species’ distribution range substantial trait variation can arise because of two fundamental drivers. First, differences in trait expression among accessions within a single species can be caused by neutral processes such as drift, migration, or lineage diversification. Second, trait diversification can be driven by selective processes in response to local biotic and abiotic environmental conditions, such as nutrient-, light- and water availability [18–20].

Detecting the signature of adaptive processes in trait variation and differentiation is a challenging task because it requires the partitioning of the observed phenotypic trait variance into genetically and environmentally based variance. In order to minimize the latter, common garden studies are often used to assess and compare trait variation across multiple provenances or genotypes. However, the expressed genetic contributions to trait variance may vary across different environments [21], limiting conclusions that can be drawn from a single common garden environment alone. Simulating different environmental conditions in a common garden experiment would hence allow a more general understanding of the observed trait variation and differentiation.

The impact of adaptive processes to genetically based phenotypic trait variation expressed among populations can be assessed by the comparison between such differentiation patterns and neutral expectations based on neutral molecular loci [22, 23]. Larger genetic differentiation in quantitative traits (*Q*_ST_) compared to neutral genetic differentiation (*F*_ST_) is interpreted as a signature of directional selection whereas the opposite case, i.e. *Q*_ST_ < *F*_ST_, indicates stabilizing selection. This approach has been employed by an increasing number of studies finding evidence for adaptation to local environments in a wide range of organisms [24]. The *Q*_ST_—*F*_ST_ approach can detect signatures of adaptation but not the environmental regimes causing trait divergence and the observed differentiation patterns. Consequently, another more qualitative approach to detect adaptive differentiation is to test for clines in mean phenotypic trait expression along environmental variables [25, 26].

Here we study quantitative trait expression and molecular genetic differentiation in response to differing nitrogen availability among a regional set of populations of the common wetland plant *Juncus effusus*, looking for signatures of adaptation. *Juncus effusus*, the ‘Common Rush’ is a perennial, self-compatible herb widely distributed in temperate wetland ecosystems [27, 28]. It is a model species for research on wetland ecosystem functioning and well characterized by a number of studies in respect to contaminant removal, e.g. on metal accumulation [29], nitrogen remediation [30, 31] and microbial activity in the rhizosphere [32]. A previous study has shown that *J*. *effusus* in Germany consists of sympatrically occurring, morphologically rather similar, but genetically highly differentiated, lineages [33]. A quantitative genetic experiment revealed strong differentiation in the expression of functional traits among few European *Juncus effusus* populations partially explained by environmental conditions of the source location suggesting a contribution of adaptive divergence [34]. To further understand functional trait expression within *J*. *effusus* and considering possible within-species lineage differentiation we ask:

1. What is the relative contribution of neutral and selective processes in quantitative trait differentiation of *J*. *effusus* and do observed patterns differ among lineages?
2. Is there a clinal differentiation of trait expression along soil environmental conditions at the regional scale, indicating adaptive responses?

## Material and methods

Between 2013 and 2014, seed families, i.e. offspring from a single maternal individual, were collected at 21 locations across central Germany and covering a wide range of habitats. *Juncus effusus* is not endangered or a protected species in Germany and no specific permissions for leaf and seed sampling were required. Spatial distance among populations ranged from 1.1 km (PWA—PWI) to 222.5 km (ZM—JEM). At each location (with population sizes varying between 20 and > 100 adult individuals), seed material was sampled from 4–6 individuals arbitrarily selected across the whole site, resulting in a total of 111 maternal individuals. Leaf material was sampled from the maternal and additional individuals resulting in a larger sample per location (N = 7–12, mean: 8.5) for molecular genetic analysis. Sampled leaf and seed material was dried with silica gel and stored in paper bags at room temperature. For each population and site, a mean seed mass was estimated by measuring length and width of seeds from 3–5 seed families by optical scanning with high resolution and applying image analysis implemented in WinSeedle (Regent Instruments Inc., Québec, Canada) and calculating a volume assuming an ellipsoid shape of the seeds. Topsoil data for each location was taken from the LUCAS topsoil dataset [35]. In order to obtain population-specific data, soil environmental variables (particle size distribution: clay-, silt- and sand content, coarse fragments, soil pH, organic- and inorganic compounds: organic carbon and carbonate, phosphorus-, nitrogen- and potassium content, and cation exchange capacity) were averaged across all data points available within a radius of 15 km of each population (1–7 per population, mean 4.8). Soil parameters obtained by this approach might not necessarily represent exact local conditions at the population origin, but rather small-scale regional patterns.

### Molecular genetic data

Genomic DNA was extracted from dried leaves using the DNeasy 96 Plant Kit (QIAGEN, Hilden, Germany) according to manufacturer’s protocol and quality was checked by gel electrophoresis and NanoDrop Spectrophotometer (ND-1000, Thermo Fisher Scientific, Wilmington, USA). Genotyping was done using 14 microsatellite loci (Jeff04, Jeff06, Jeff10, Jeff11, Jeff29, Jeff36, Jeff52, AY493568, AY493569, Jeff058, Jeff059, Jeff067, Jeff069, Jeff074) previously described by Michalski and Durka [33], Michalski and Durka [36] and two additional loci (Jeff111 and Jeff115, S1 Table). PCR fragments were obtained using directly fluorescent-labeled primers or primers with universal fluorescent-labeled M13R and CAG tails [37]. Amplification reactions with directly fluorescent-labeled primers were carried out in a volume of 8 μl containing 1 μl genomic DNA (~ 20 ng / μl), 4 μl QIAGEN Multiplex Mastermix, 1.2 μl RNase-free H_2_O, 0.8 μl QIAGEN 5 × Q-Solution and 0.2 μM of each forward and reverse primer. PCR conditions were as follows: 95 °C for 15 min; followed by 35 cycles of 95 °C for 30 s; 58 °C for 40 s and 72 °C for 1 min, followed by 72 °C for 15 min. The amplification with M13R or CAG tailed primers was performed in a 5 μl reaction mixture that contained 1.0 μl genomic DNA (~ 20 ng / μl), 2.5 μl QIAGEN Multiplex Mastermix, 0.05 μM of the M13R or CAG tagged primer, 0.25 μM of forward and reverse primer mix and 0.25 μM of fluorescently labelled M13R or CAG primer. A touchdown PCR was run as follows: 95 °C for 15 min; followed by 20 cycles (94 °C for 30 s, 60 °C for 30 s with decrease of 0.5 °C per cycle, 72 °C for 90 s), followed by 20 cycles (94 °C for 30 s, 50 °C for 30 s, 72 °C for 90 s) and close with 72 °C for 10 min. Fragments were run on an ABI 3130 (Applied Biosystems, Foster City, California, USA) with size standard LIZ 500 (Applied Biosystems) and scoring was done using GeneMapper 5 (Applied Biosystems).

### Quantitative genetic data

Trait expression in response to differing nitrogen availability was quantified in a common garden experiment in the experimental field station Bad Lauchstädt (51°23’N, 11°52’E). In December 2014, seeds were germinated in quickpots (96 cells, 4 cm diameter x 8 cm deep; Hermann Meyer KG, Rellingen, Germany) filled with a 2:1 (vol/vol) mixture of commercial soil (Fruhstorfer soil type P) and sand using a climate chamber with a 12 h photoperiod and mean day and night temperature of 30 °C and 20 °C, respectively. In April 2015, seedlings of approximately the same size were transplanted into 3 l pots (17 cm diameter x 18.5 cm deep) filled with a mixture of commercial soil and sand (2:1, vol/vol; Fruhstorfer soil type P). Sample extracts (N = 10) from 10 g air-dried soil mixture suspended in 40 ml 1 M potassium chloride gave average concentrations of 49.3 mg / kg NO^3-^—N and 0.45 NH4+—N mg / kg detected by flow-rate injection analyzer FIAstar^™^ 5000 Analyzer (FOSS Analytical, Denmark). All individuals were constantly watered and after one month of transplanting plants received either no or additional fertilizer of weekly doses of dissolved ammonium nitrate (NH_4_NO_3_), equivalent to a total of 72 kg N ha^-1^ yr^-1^ and 153 kg N ha^-1^ yr^-1^, respectively. Thus, the fertilization treatment consistent of three levels: (1) a low nitrogen load with no additional nitrogen (T0), (2) a moderate nitrogen load (T70) and (3) a high nitrogen load (T150). Because of a low initial seed number and insufficient germination- and establishment rate for a number of populations, not enough offspring could be raised to ensure a fully balanced design (Table 1). Pots were arranged randomly in the greenhouse.

**Table 1:** Location of studied *Juncus effusus* populations, sample sizes used in the common garden experiment, lineage membership according to STRUCTURE and expected heterozygosity (*H*_e_).

ID	Population	Latitude (°N)	Longitude (°E)	No. of seed families / total no. of individuals			Population structure Q values			*H*_e_
				T0	T70	T150	Eff1	Eff2	Eff3	
BB	Brandberge	51.5117	11.9263	4 / 16	-	4 / 12	**0.975**	0.023	0.003	0.359
DB	Drübeck	51.8531	10.7057	5 / 14	-	-	0.116	**0.877**	0.007	0.356
EB	Ettersberg	51.0320	11.2638	5 / 20	4 / 11	4 / 12	0.001	**0.996**	0.003	0.181
ES	Esperstedt	51.4192	11.6676	6 / 24	4 / 12	4 / 12	0.001	**0.998**	0.001	0.042
ET	Elendstal	51.7475	10.6810	6 / 24	4 / 12	4 / 12	0.001	0.002	**0.997**	0.133
GF	Gräfenroda	50.7438	10.7914	6 / 24	4 / 12	4 / 12	**0.997**	0.002	0.001	0.256
GR	Groß Rosenburg	51.9124	11.9178	5 / 14	-	-	0.002	**0.994**	0.004	0.144
JAM	Jävenitzer Moor	52.5029	11.4720	5 / 19	4 / 12	4 / 12	**0.986**	0.003	0.011	0.275
JEM	Jemmeritzer Moor	52.6365	11.2620	6 / 24	4 / 12	4 / 11	**0.989**	0.009	0.002	0.302
MS	Massanei	51.0587	13.0458	5 / 20	-	4 / 12	0.005	**0.989**	0.006	0.075
OH	Oberhof	50.7150	10.7769	5 / 17	-	4 / 12	**0.993**	0.005	0.002	0.197
PWA	Pressel (forest)	51.5739	12.7381	5 / 20	4 / 12	4 / 12	**0.924**	0.022	0.055	0.341
PWI	Pressel (meadow)	51.5655	12.7322	5 / 20	4 / 12	4 / 12	0.021	**0.941**	0.038	0.194
RB	Rappbodetalsperre	51.7407	10.8875	5 / 16	-	-	0.002	0.002	**0.996**	0.027
RO	Rösa	51.6114	12.4493	5 / 20	4 / 12	4 /12	0.003	**0.981**	0.017	0.203
SC	Schierke	51.7732	10.6388	6 / 22	-	-	0.002	0.002	**0.996**	0.250
SF1	Siptenfelde	51.6605	11.0484	4 / 16	-	-	**0.996**	0.002	0.002	0.188
SF2				5 / 20	-	-	0.002	**0.997**	0.001	0.050
WD	Wermsdorf	51.3017	12.9030	4 / 15	-	4 / 12	0.001	**0.988**	0.011	0.144
WL	Wörlitz	51.8336	12.4348	5 / 20	4 / 12	4 / 12	0.024	**0.967**	0.010	0.181
ZM	Zella Mehlis	50.6722	10.6739	5 / 20	4 / 12	4 / 12	**0.997**	0.001	0.002	0.000
ZR	Ziegelroda	51.3455	11.4931	4 / 14	4 / 12	4 / 12	0.002	**0.996**	0.002	0.198

At the start of the experiment, initial plant biomass was indirectly assessed as plant height (H), i.e. the mean height of the two longest leaves, times the number of stems (S). We repeated this measurement at the end of experiment to estimate a relative growth rate (RGR = (H*S)end − (H*S)_start_) / (H*S)_start_). Ten weeks after initiating the treatment, all plants were harvested. For each individual, the weight of the above- (AGBM) and belowground biomass (BGBM) was determined after drying at 60 °C for 48 h. A root to shoot ratio was calculated as the quotient between BGBM and AGBM. To assess the biomass quality, we calculated leaf dry matter content (LDMC) as dry divided by fresh mass.

Two individuals per seed family and treatment were randomly selected for chemical analyses. A representative fraction (~ 10 mg) of above- and belowground biomass was milled and C and N concentrations were measured using a CHNS/O elemental analyzer (Vario EL III, Element Analyzer, Elementar, Hanau, Germany). From these data, we calculated C:N ratios for above- (AG-C:N) and belowground biomass (BG-C:N) as well as total aboveground N accumulation (AG-N) as N content per g leaf tissue multiplied by the dry weight. The same subset of individuals was also used for pH measurement of the pot soil using the method described by Hoffmann [38]. Air-dried and sieved (2 mm) soil samples were suspended in a ratio 1:2.5 with 0.01 M CaCl_2_ solution and analyzed using a pH Meter (Knick pH-Meter 766 Calimatic, Berlin, Germany). Root porosity was estimated using the microbalance method described in Visser and Bögemann [39]. Eight weeks after the start of the experiment, we extracted for each individual three root tips. Ten mm behind the root apex, a 30 mm long root segment was excised and surface water was carefully dried with tissue paper and the weight of the root sample was determined. Samples were then placed in a water-filled 1.5 ml Eppendorf tube and put under vacuum conditions for 5 min and infiltrated root segments were weighed again. Root porosity was calculated as the weight difference relative to the weight after infiltration and multiplied by the specific weight for *J*. *effusus* roots given in Visser and Bögemann [39]. The three values obtained were averaged to obtain an individual estimate.

### Genetic diversity and population structure

For the microsatellite data, we used a principal coordinate analysis (PCoA) computed in GenAlEx [40] to visualize genetic similarities among *J*. *effusus* individuals (S1 Fig). Population structure was determined using a Bayesian model-based clustering approach implemented in STRUCTURE 2.3.4 [41–43]. An admixture model with correlated allelic frequencies was used to determine the most likely number of clusters (K). We used 10 independent runs each with 150,000 iterations of which 50,000 were discarded as burn-in. The optimum K was identified using the approach of Evanno *et al*., [44] implemented in the web program STRUCTURE HARVESTER 0.9.94 [45]. Individuals were assigned to lineages based on individual assignment probabilities (Q values > 0.7). For each population, expected heterozygosity *H*_e_ [46] and global genetic differentiation (*F*_ST_, [46]) among populations and lineages identified above was calculated using FSTAT ([47], Version 2.9.3.2).

### Data analyses

To assess the quantitative trait expression in response to treatment and lineage, we used a linear mixed effect model implemented in package ‘lme4’ [48] for R [49] explaining the observed variation in each trait separately by N supply, lineage identity, and the lineage × treatment interaction as fixed effects, and seed family nested in origin and their interaction with treatment as random effects. Subsequently we did post hoc analyses to test for pairwise differences among treatments and lineages within treatment.

Each quantitative trait genetic differentiation among populations was estimated as *Q*_ST_ = V_AP_ / (V_AP_ + 2 V_WP_) [50], by partitioning the total phenotypic variance into variance among- (V_AP_) and within (V_WP_) populations, with the latter given by the seed family component. Variance components were derived from generalized linear mixed models using a Bayesian framework implemented in the package ‘MCMCglmm’ [51] for R [49]. Bayesian approaches have been recommended for *Q*_ST_ estimation as they allow for flexible experimental designs and return mostly unbiased precision estimates [52, 53]. For each trait we calculated (1) an across-treatment *Q*_ST_ for all populations using models including treatment as fixed, and population and seed family as random effects, and (2) treatment-specific *Q*_ST_ values with only population and seed family as random effects. And (3) we calculated lineage-specific across-treatment *Q*_ST_ values. For (3), *Q*_ST_ values were calculated for only two out of three genetic lineages found (see below), as sample size for the third lineage was very low (see Results). Credibility intervals for all estimates were directly taken from the posterior distribution.

To test whether quantitative genetic differentiation in mean traits (*Q*_ST_) differed from neutral expectations, we followed the approach of Whitlock and Guillaume [54] by reporting the difference between the observed *Q*_ST_ and a simulation expected under neutrality (*Q*^n^_ST_). A significant positive or negative deviation from zero directly indicates directional or stabilizing selection, respectively. The distribution of *Q*^n^_ST_ values was calculated by simulating a neutral among-population variance 1000 times as V^n^_P_ = *F*_ST_ * (2 * V_A_ / (1 − *F*_ST_)) and then multiplying by a factor r / (n_pop_ − 1) with n_pop_ the number of populations considered and r being a random number drawn from a chi^2^—distribution with npop − 1 degrees of freedom, to simulate the sampling distribution around this expectation. For each simulation, the *F*_ST_ value for the respective set of populations was used and a V_A_ value was sampled from the posterior distribution of the models described above. *Q*^n^_ST_ values were then computed using the observed within-population variance. The test statistic was calculated as the difference between 1000 *Q*_ST_ values drawn from the posterior distribution of the model and 1000 simulated *Q*^n^_ST_ values and considered to be significant if the 95% credible interval did not include zero.

In order to assess whether variability and differentiation in trait expression relates to soil environmental characteristics of population origin, we performed several analyses. (A) Soil environmental parameters were reduced by applying a principal component analysis (PCA) on standardized soil parameters using function prcomp() from ‘stats’ package for R [49]. Then, the first two axes scores accounting for the majority of variation (43 and 30% of the total variation, respectively; S2 Fig) were used separately to explain trait expression in response to (1) soil environment of the region of origin in dependence of experimental N supply and (2) soil environment in dependence of intraspecific lineage affiliation for each treatment conditions separately to test for potential lineage-specific patterns. For (1) we used linear mixed effect models with PCA axis scores and experimental treatment as fixed and lineage, population, and seed family as random effects to account for repeated measurements. For (2) we calculated models for each treatment condition separately explaining individual trait variation by PCA axis scores, lineage, and their interaction as fixed and population and seed family as random effects. To account for possible maternal effects mediated by seed size [55], we repeated all analyses using mean seed mass per population as covariate. Additionally, we tested for relations between expected heterozygosity (*H*_e_) and first axis scores of soil parameters. (B) Variation in pairwise trait differentiation (*Q*^ij^_ST_) between populations was explained by pairwise environmental distances jointly considering neutral genetic differentiation (*F*^ij^_ST_) using a multiple matrix regression approach [56]. Environmental distances were calculated as Euclidean distances using standardized soil parameters. Trait and molecular genetic differentiation matrices were standardized prior to the analyses in order to allow comparisons of coefficients. Significances for individual regression coefficients were assessed by comparing observations against a null distribution obtained by permuting the dependent matrix 9999 times. This approach was applied to explain (1) across-treatment trait differentiation with the subset of populations present in all treatment conditions (N = 12), and (2) for each treatment condition separately and (3) for each lineage and treatment condition separately.

## Results

### Genetic diversity and population structure

**Fig 1:**
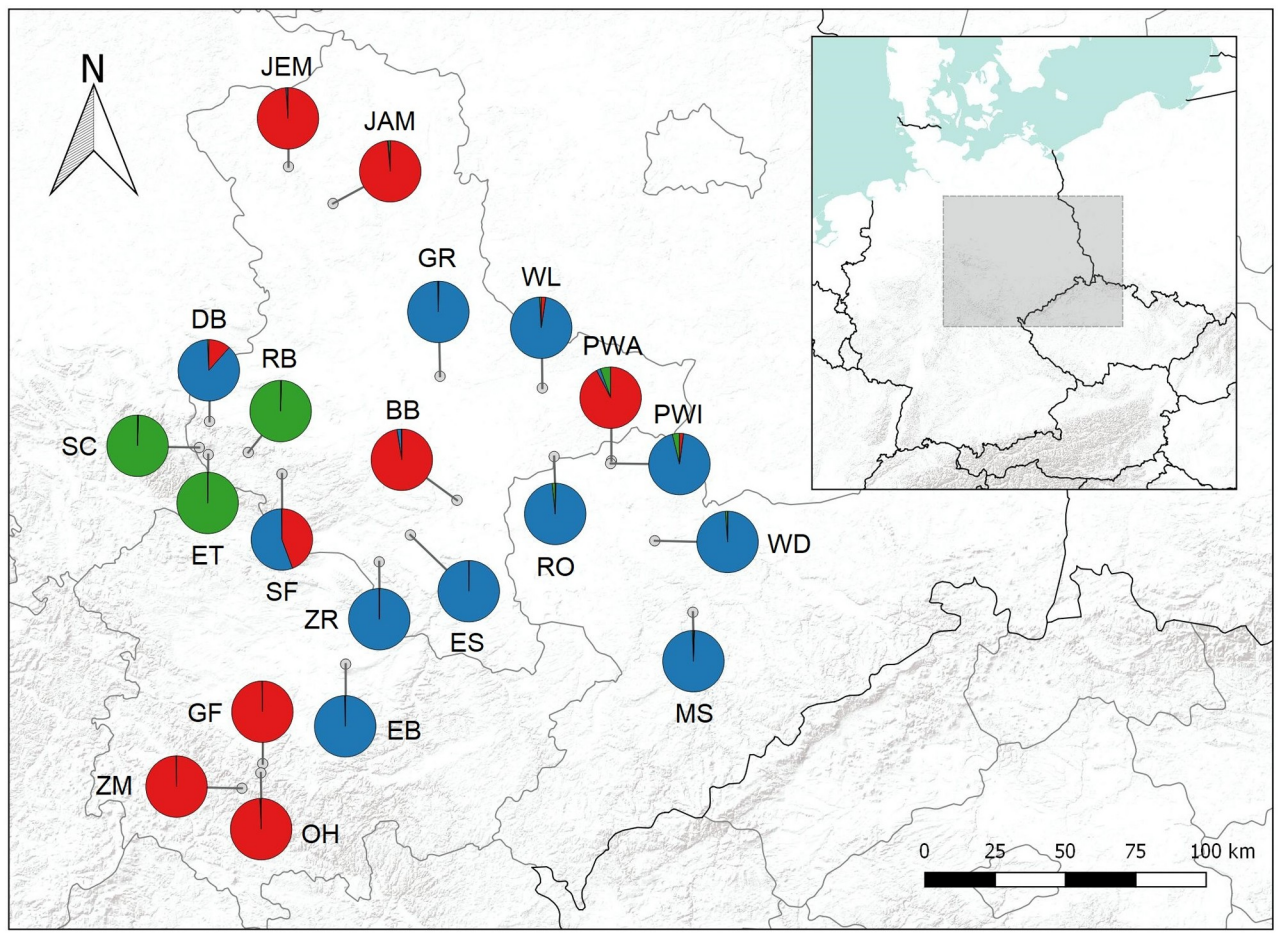
Locations of the 21 sampled *Juncus effusus* populations and, in color, lineages membership (red: Eff1, blue: Eff2, green: Eff3). For population acronyms see Table 1.

In a total of 111 individuals, we found 74 different alleles at 16 different microsatellite loci. The expected heterozygosity varied substantially among populations (mean *H*_e_ = 0.187, SD = 0.089; Table 1). Genetic differentiation among populations was very pronounced (global *F*_ST_ = 0.66; SD = 0.120). Bayesian cluster analysis revealed a single most likely solution with samples forming three distinct genetic lineages (K = 3, ΔK = 273.4) subsequently described as lineages Eff1, Eff2 and Eff3, with the latter represented by individuals of only three populations (Fig 1). All individuals could be unambiguously assigned to either lineage (Q > 0.7). Only one location showed a strong admixture (SF) and samples from this location were assigned to either Eff1 or Eff2 and thus, samples were considered as two populations in the following analyses. Between lineage differentiation was substantial with *F*_ST_^Eff1-Eff2^ = 0.405, *F*_ST_^Eff1-Eff3^ = 0.372 and *F*_ST_^Eff2-Eff3^ = 0.412. Within lineages, genetic differentiation among populations was still very pronounced with *F*_ST_ = 0.289, 0.385 and 0.614 for Eff1, Eff2 and Eff3, respectively.

### Effect of N addition on plant trait expression

**Fig 2:**
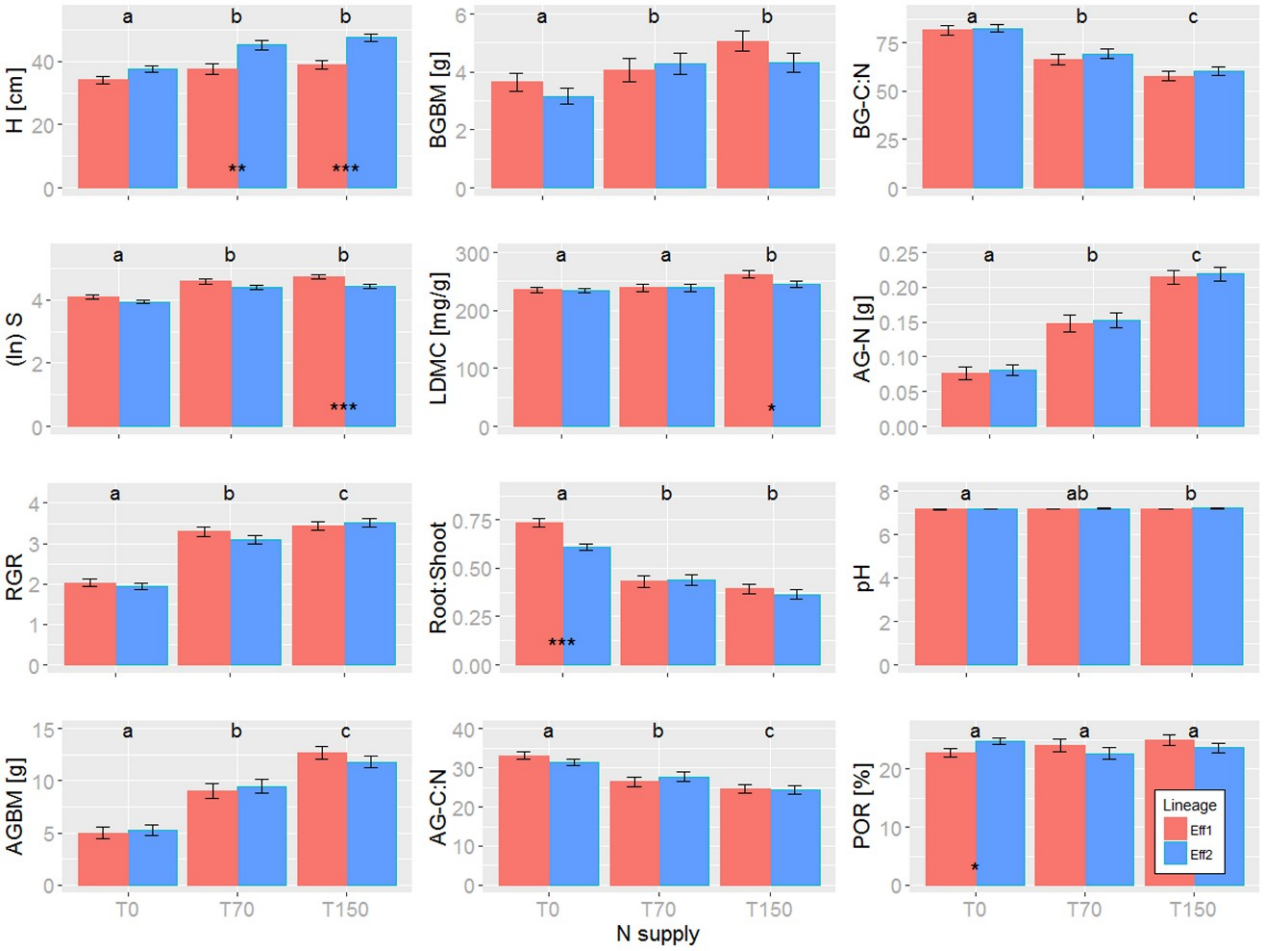
Quantitative trait expression in response to treatment and lineage membership in *Juncus effusus*. Different letters indicate significant differences among N supplies and asterisks indicate significantly differences (α < 0.05) among lineages (red: Eff1, blue: Eff2) within treatments. H: plant height; S: number of stems; RGR: relative growth rate; AGBM: aboveground biomass; BGBM: belowground biomass; LDMC: leaf dry matter content; Root:Shoot: ratio root to shoot; AG-C:N: carbon to nitrogen ratio of aboveground biomass; BG-C:N: carbon to nitrogen ratio of belowground biomass; AG-N: total aboveground N accumulation; pH: soil pH; POR: root porosity.

The overall effect of N addition and lineage on trait expression was assessed for individuals from lineages Eff1 and Eff2 only as sample size for Eff3 was too low. In general, all investigated quantitative traits responded significantly to the N addition treatment, except for root porosity (Fig 2). As expected traits such as plant height, number of stems, relative growth rate, above- and belowground biomass as well as aboveground N increased under N supply, whereas above- and belowground C:N and Root:Shoot decreased (Fig 2). Differences between lineages were inconsistent across treatment conditions and mostly expressed in vegetative characters such as plant height and number of stems (Fig 2). The complete data set is available in S1 Appendix.

### Quantitative genetic divergence

**Fig 3:**
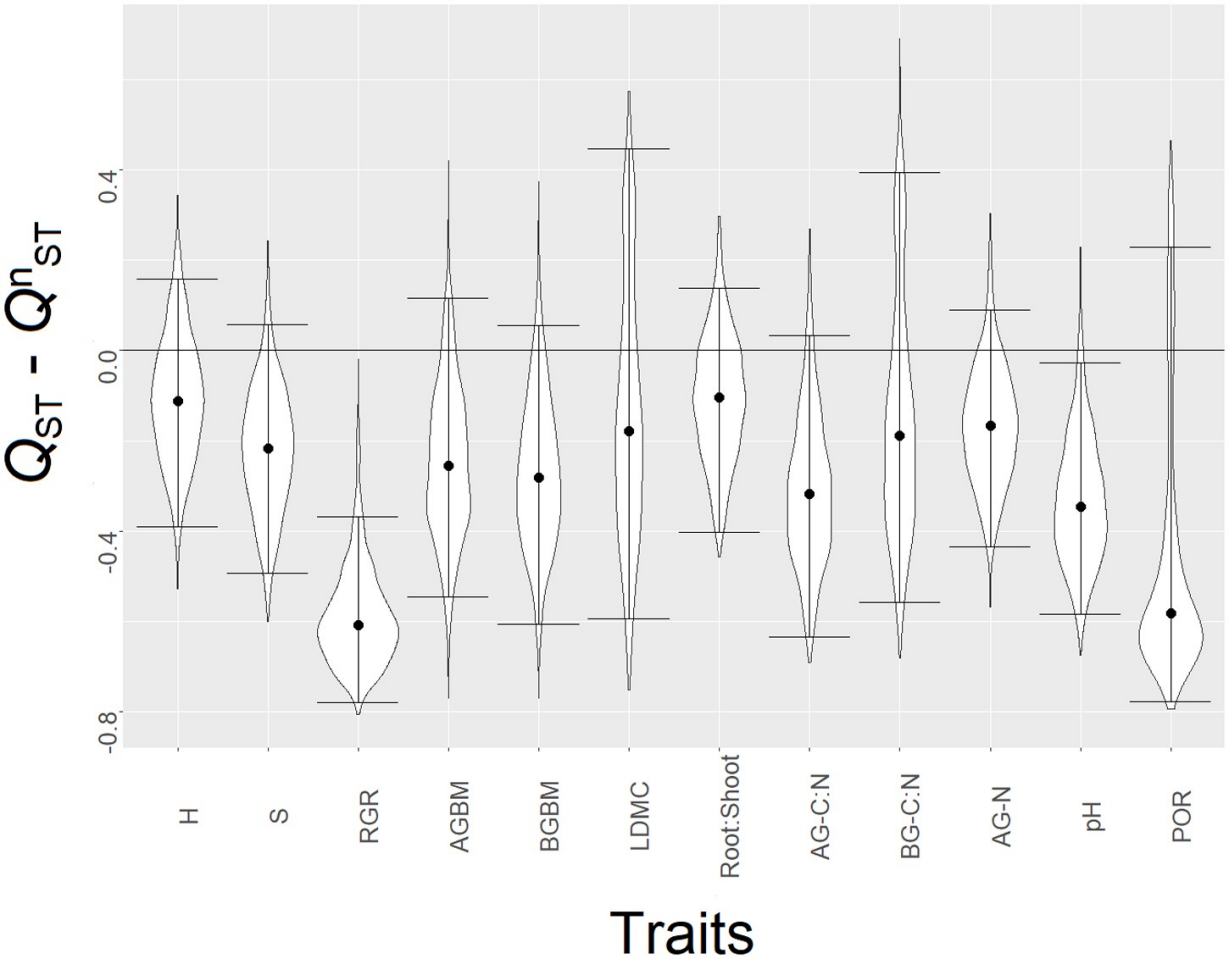
Violin plots showing the comparison between quantitative genetic differentiation among populations (*Q*_ST_) across treatment conditions and a neutral expectation (*Q*^n^_ST_) for all measured plant traits. Dots indicate the posterior median of the difference and bars the 95% credibility interval. A signature of directional or balancing selection is indicated by a significant deviation from the zero expectation. For trait explanations see Fig 2.

Quantitative genetic differentiation in mean traits across treatment conditions ranged from *Q*_ST_ = 0.021 to 0.548 and was substantial for most traits except for relative growth rate, AG-C:N and root porosity (S2 Table). Genetic differentiation was inconsistently expressed when treatment conditions were treated separately, most notably for AG-C:N, BG-C:N, above- and belowground biomass, but without a clear trend towards less or more differentiation at a specific N addition level (S2 Table). Genetic differentiation differed between lineages strongly for plant height (Eff1: *Q*_ST_ = 0.699, Eff2: *Q*_ST_ = 0.221) and LDMC (Eff1: *Q*_ST_ = 0.992, Eff2: *Q*_ST_ = 0.067), whereas for most other traits differentiation patterns were more similar (S2 Table). The observed genetic differentiation for the measured traits did not significantly exceed neutral expectations when compared across treatments or for each treatment and lineage separately (Fig 3, S3 and S4 Figs). Differentiation lower than expected under neutrality was found in several comparisons, most often for relative growth rate but also for soil pH, root to shoot ratio or aboveground N accumulation.

Expected heterozygosity at population level significantly increased with PCA scores of the first axis only (r = 0.654, P < 0.05), which mainly represented sand and clay content and cation exchange capacity.

### Trait clines with soil environments

**Fig 4:**
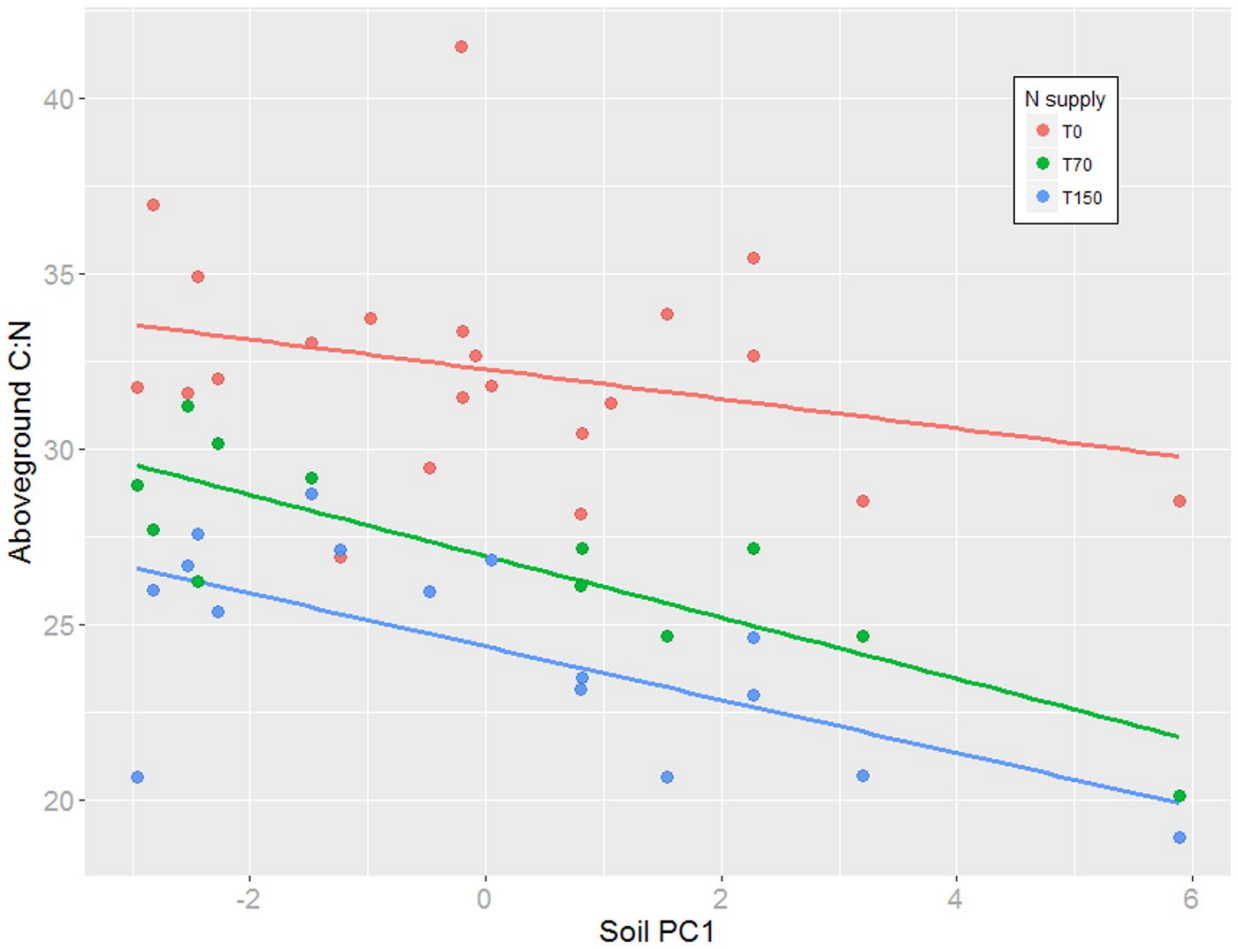
Correlation between aboveground C:N ratio and soil environmental parameters of population origin (factor scores for the first axis of a PCA on all soil parameters) in dependence of experimental N supply. Note that for visualization only population mean values are plotted. Colors represent the different nitrogen addition levels applied.

Overall mean trait expression at the population level varied with soil environmental variation expressed as PCA axis 1 for above- and belowground C:N ratio (Fig 4), number of stems as well as LDMC and belowground biomass (S3 Table). Overall, trait expression did not co-vary with PCA axis 2. These results were not altered by the inclusion of seed mass as covariate (data not shown). Significant differences between lineages Eff1 and Eff2 were present for some relationships, e.g. slopes of the clines along PCA axis 1 differed for plant height, aboveground N and pH in the pot soil, but were not consistently expressed across the different N addition levels (S4 and S5 Tables).

Pairwise genetic trait differentiation across treatments increased significantly with distance in soil environment for aboveground C:N, whereas differentiation in plant height and number of stems mirrored neutral genetic differentiation (S6 Table). A similar pattern was found when treatment conditions were analyzed separately. Treating each lineage separately within treatment conditions, only a few significant correlations could be found which were also inconsistently expressed among treatment conditions and the two lineages considered (S7 Table).

## Discussion

Supporting recent findings by Michalski and Durka [33], our results show that in Central Europe *Juncus effusus* consists of multiple, genetically well separated lineages that partly co-occur at the same location but show only limited differences in trait expression. It has been suggested, that the co-existence of these genotypic lineages is a result of an allopatric origin with secondary contact and a divergence in flowering phenology could contribute to the maintenance of sympatric lineages [33].

### Effect of N addition on plant trait expression

Nitrogen addition increased plant height, number of stems, relative growth rate as well as above- and belowground biomass in *J*. *effusus*, which can be expected from previous studies investigating the effect of N fertilization [57, 58]. Furthermore, our results showed that N addition increased total aboveground N accumulation and decreased the C:N ratio in leaves and roots, as was also observed in several other studies [59, 60]. Soil N availability stimulates plant growth and N incorporation into biomass which is well known for terrestrial plant species [61]. Surprisingly, in our study *J*. *effusus* showed a weak but significantly higher LDMC under the highest N availability compared to the other treatment conditions. In general, LDMC correlates negatively with relative growth rate [62] which in turn is positively affected by increased N availability as shown by our results (but see e.g. [59]). Often, light competition induced by N enrichment will decrease LDMC and in turn may increase the SLA (‘specific leaf area’). However, all individuals experienced similar sunlight conditions and light competition because treatments were applied arbitrarily across pots. Furthermore, we found a greater above- than belowground growth under N addition indicated by a significantly lower root to shoot ratio in N treatments. Changes in allocation in response to N concentration was expected and consistent with other studies [63]. N limitation may stimulate root growth to increase nutrient uptake and N addition may lead to a shift of N from belowground biomass to leaf biomass because of a N demand for physiological activities in leaves, e.g. for competition for light [61]. Higher root porosity under low N conditions can increase the remobilization of nutrients [11] and thus, improve N acquisition and plant growth under N limitation [64, 65]. However, compared to similar experiments on wetland macrophytes, in our study root porosity of *J*. *effusus* did not significantly decrease with N addition (cf. Born and Michalski [34]).

### Quantitative genetic divergence

We found high differentiation among populations and lineages at molecular and quantitative trait levels, confirming earlier results [33, 34], which are probably related to the life history of *J*. *effusus*. The species is an efficient pioneer species and colonizer due to a fast growth rate [66], high seed production [67] and a predominantly selfing mating system [33, 68]. Both frequent founder events and selfing mating system are expected to reduce effective population size and increase genetic drift effects. Consequently, at the population-level, genetic variation can be reduced and associated with strong genetic differentiation among populations [69].

Indeed, *Q*_ST_-estimates for *J*. *effusus* under N addition were exceptionally high for some traits such as aboveground C:N (T70: *Q*_ST_ = 0.886) and belowground C:N (T70: *Q*_ST_ = 0.860, T150: *Q*_ST_ = 0.941). Similarly, populations of the predominantly selfing *Senecio vulgaris* showed a strong degree of quantitative trait divergence for growth and life history traits (*Q*_ST_ = 0.26–0.77, Steinger *et al*., [70]). The partially self-fertilizing species *Arabis fecunda* even showed an average *Q*_ST_ of 0.94 for morphological traits [71]. A meta-analysis of quantitative trait divergence revealed that the vast majority of the *Q*_ST_ values typically exceeds neutral expectations based on molecular markers, indicating a predominant role of divergent selection in shaping quantitative trait differentiation [24]. In our study, the average *Q*_ST_ of 0.36 across all populations and treatments was comparable to the average of *Q*_ST_ = 0.35 reported by Leinonen *et al*., [24]. However, when compared to neutral expectations, signatures of adaptive differentiation could not be found for any of the traits assessed in our study. For most traits, differentiation did not differ from neutral expectations and for some traits (e.g. soil pH or relative growth rate) differentiation patterns instead showed evidence for stabilizing selection (*Q*_ST_ < *Q*^n^_ST_). Neutral differentiation and stabilizing selection as causes for quantitative trait differentiation is often found for rare species with small population sizes and a high level of habitat fragmentation and isolation e.g. *Liatris scariosa* [72], *Primula sieboldii* [73] and *Psilopeganum sinense* [74]. It has been further argued that the ecological niche of rare species is restricted, causing a homogenous selection pressure, resulting in a relatively low quantitative trait divergence [75]. However, the ecological niche and distributional range of *J*. *effusus* is rather broad, questioning the importance of stabilizing selection for trait expression in this species.

Quantitative trait differentiation estimated by *Q*_ST_ can be biased, possibly limiting the conclusions that can be drawn from *Q*_ST_—*F*_ST_ comparisons. First, maternal effects may affect trait expression in general and can bias *Q*_ST_ estimates downwards [76], which has to be considered particularly for early life traits like initial growth and survival [77]. In our study, the majority of traits were measured at the end of the growing season on adult individuals reducing the probability of such a bias. Still, maternal effects cannot be ruled out completely, as shown by the effects of seed size on treatment specific trait clines (S4 and S5 Tables). Second, non-additive genetic effects may also decrease *Q*_ST_ estimates possibly resulting in *Q*_ST_ < *F*_ST_ outcomes without stabilizing selection. Whereas dominance effects for inbred species such as *J*. *effusus* might be of less importance [78], epistatic and pleiotropic effects may introduce a bias which is often neglected in the *Q*_ST_—*F*_ST_ approach. However, for our study this bias is not very likely as it would lead to false positive signatures of adaptive divergence only. The use of microsatellite data for *Q*_ST_—*F*_ST_ comparisons has been criticized because the potentially large number of alleles may result in downwardly biased *F*_ST_ estimates [79]. Instead, the use of SNP data has been recommended. Indeed, preliminary results for SNP genotyping using the same set of populations and individuals and a small set of loci (N = 32) resulted in a higher overall *F*_ST_ estimate (*F*_ST_ = 0.823 (SNP) vs. *F*_ST_ = 0.660 (Microsatellite); Born, unpublished data). This marker bias could query signatures of diversifying selection, which, however, were not found here.

### Trait clines with soil environments

Genetically based phenotypic trait variation along environmental or geographical clines has been reported for many plant species [80–82] and is considered to be a signature of adaptation (but see [83]). Whereas a plant’s response to climatic conditions, latitude or elevation of origin is frequently studied, the impact of soil properties on adaptive trait expression has been much less investigated [84]. Here, we found that at the population-level, mean trait expression as well as pairwise trait differentiation for several traits (e.g. AG-C:N, Root:Shoot ratio or plant height) correlated significantly with soil environmental data of the site of origin and distances, respectively, suggesting adaptive trait variation in response to soil characteristics.

However, these correlations were not consistently found for these traits when data of the different N concentrations applied was analyzed separately, suggesting that in different environments the (genetic) basis for trait expression can differ substantially [85]. In the two N addition treatments, most consistently aboveground C:N ratio showed patterns of adaptive trait variation and differentiation. Plants originating from poorer, sandier soils with less capacity to hold exchangeable cations (CEC) expressed significantly higher C:N ratios as compared to plants from more fertile soils. Indeed, it is known from field observations that the aboveground C:N ratio of herbaceous plants increases when nutrient availability in the soil becomes more limited [86]. Such responses have been explained by ecophysiological mechanisms of carbon (re-)allocation [87]. Our findings suggest that these mechanisms are not purely plastic for *J*. *effusus* [cf. 80], but are selectively modified by local soil conditions.

We found only little and inconsistent evidence for selective mechanisms to vary between the lineages within *J*. *effusus* which would be indicated by significant interactions between lineage and soil environmental conditions of origin when explaining population mean traits. This would support the idea that the lineages within *J*. *effusus* are the result of neutral divergence following e.g. variance [33].

In summary, increased effects of genetic drift and limited gene flow for the selfing colonizer *J*. *effusus* resulted in a very pronounced neutral genetic differentiation with few differences between lineages within the species. Adaptive trait differentiation in response to soil environmental conditions might still be present as indicated by significant trait clines but could not be detected by *Q*_ST_—*F*_ST_ comparisons.

## Supporting information

S1 Appendix Dataset of all studied individuals and their quantitative traits in response to nitrogen availability. (XLSX)

S1 Table Characteristics of two newly developed microsatellite markers in *Juncus effusus* including repeat motif, primer sequence for forward- and reverse primer, reaction mixture using universal fluorescent-labeled tailed primers, allelic size range and accession number in gene bank. (DOCX)

S2 Table Quantitative trait divergence (*Q*_ST_) among studied *Juncus effusus* populations across treatments, for the different N addition level (T0, T70 and T150) and for lineages Eff1 and Eff2 across treatments separately. (DOCX)

S3 Table Effects of soil environment of the source location (measured as the first and second axis of a principal component analysis of all soil parameters, PC1 and PC2) and experimental nitrogen addition and their interaction on mean quantitative trait expression in *Juncus effusus*. (DOCX)

S4 Table Effects of soil environment of the source location (measured as the first axis of a principal component analysis of all soil parameters, PC1) and lineage membership and their interaction on mean quantitative trait expression in *Juncus effusus*. (DOCX)

S5 Table Effects of soil environment of the source location (measured as the second axis of a principal component analysis of all soil parameters, PC2) and lineage membership and their interaction on mean quantitative trait expression in *Juncus effusus*. (DOCX)

S6 Table Multiple matrix regression with randomization analysis (MMRR) explaining pairwise trait differentiation between populations (*Q*ijST) jointly by (A) pairwise soil environmental distance and (B) pairwise neutral genetic differentiation (*F*ijST) for the subset of 12 *Juncus effusus* populations across treatment conditions and for each treatment T0 (N = 22), T70 (N = 12) and T150 (N = 16) separately. (DOCX)

S7 Table Multiple matrix regression with randomization analysis (MMRR) explaining pairwise trait differentiation between populations (*Q*ijST) jointly by (A) pairwise soil environmental distance and (B) pairwise neutral genetic differentiation (*F*ijST) for the lineages Eff1 and Eff2 and treatment conditions (T0, T70 and T150) separately. (DOCX)

S1 Fig Principal coordinate analysis (PCoA) plot based on pairwise genotypic distances among individuals. Circles represent individuals grouped in three distinct clusters indicated by colors (red: Eff1, blue: Eff2, green: Eff3).
(DOCX)

S2 Fig The Principal component analysis (PCA) of 11 soil variables for 22 *Juncus effusus* populations. Grey arrows indicate loadings of each soil variable on the two axes (particle size distribution: clay-, silt- and sand content, coarse fragments, pH (CaCl_2_), organic carbon (OC), potassium- (K), phosphorus- (P), carbonate- (CACO_3_) and total nitrogen (N) content and cation exchange capacity (CEC)), while colored symbols represent populations and their underlying genotype (red: Eff1, blue: Eff2, green: Eff3) in the environmental space.
(DOCX)

S3 Fig Violin plot shows the comparison of quantitative genetic divergence (*Q*_ST_) and with the expected distribution under neutrality (*Q*^n^_ST_) for each treatment separately (A: T0, N = 22; B: T70, N = 12 and C: T150, N = 16). (DOCX)

S4 Fig Violin plot shows the comparison of quantitative genetic divergence (*Q*_ST_) and with the expected distribution under neutrality (*Q*^n^_ST_) within detected lineages Eff1 (A, N = 8) and Eff2 (B, N = 11). (DOCX)
