# Supplementary material for: Trait expression and signatures of adaptation in response to nitrogen addition in the common wetland plant Juncus effusus
Source: PLoS One. 2019 Jan 4;14(1):e0209886. doi: 10.1371/journal.pone.0209886 (PMC6319709; doi:10.1371/journal.pone.0209886)
Supplement: S1 Table — (DOCX) [file pone.0209886.s002.docx]

**S1 Table. Characteristics of two newly developed microsatellite markers in *Juncus effusus* including repeat motif, primer sequence for forward- and reverse primer, reaction mixture using universal fluorescent-labeled tailed primers, allelic size range and accession number in gene bank.**

| Locus | Repeat  motif | Primer sequence (5’ – 3’) | Reaction mix | Size range (bp) | Label | Accession number |
| --- | --- | --- | --- | --- | --- | --- |
| Jeff111 | CT(6) | F: CAGTCGGGCGTCATCAGAATCTTCCTCCTCTGAC  R: GTTTAGAGAGAAACAGCAGCACTC | CAG | 336 - 372 | PET-F | MH205689 |
| Jeff115 | AAAT(6) | F: GTTTGGGTATACATGAAATTCCAC  R: GGAAACAGCTATGACCATGGAGATGATATGCGACTC | M13R | 114 - 131 | VIC-R | MH205690 |
