# Supplementary material for: Trait expression and signatures of adaptation in response to nitrogen addition in the common wetland plant Juncus effusus
Source: PLoS One. 2019 Jan 4;14(1):e0209886. doi: 10.1371/journal.pone.0209886 (PMC6319709; doi:10.1371/journal.pone.0209886)
Supplement: S2 Table — (DOCX) [file pone.0209886.s003.docx]

**S2 Table. Quantitative trait divergence (*Q*_ST_) among studied *Juncus effusus* populations across treatments, for the different N addition level (T0, T70 and T150) and for lineages Eff1 and Eff2 across treatments separately.**

| Functional traits | *Q*_ST_ | *Q*_ST_ | *Q*_ST_ | *Q*_ST_ | *Q*_ST_ | *Q*_ST_ |
| --- | --- | --- | --- | --- | --- | --- |
|  | (across treatments) | (T0) | (T70) | (T150) | (Eff1) | (Eff2) |
| H | **0.548** | **0.946** | **0.684** | **0.677** | 0.699 | 0.221 |
| S | **0.429** | **0.521** | 0.624 | **0.573** | 0.346 | 0.332 |
| RGR | 0.021 | 0.240 | 0.005 | 0.027 | 0.001 | 0.062 |
| AGBM | **0.388** | **0.768** | 0.651 | 0.227 | **0.716** | 0.402 |
| BGBM | **0.363** | **0.462** | **0.740** | 0.273 | 0.419 | 0.521 |
| LDMC | **0.463** | **0.872** | 0.560 | 0.905 | **0.992** | 0.067 |
| Root:Shoot | **0.545** | **0.436** | 0.205 | 0.295 | 0.350 | 0.296 |
| AG-C:N | 0.326 | 0.230 | **0.886** | **0.416** | 0.261 | 0.445 |
| BG-C:N | **0.444** | 0.194 | **0.860** | **0.941** | 0.544 | 0.821 |
| AG-N | **0.488** | **0.221** | 0.179 | 0.230 | **0.455** | **0.553** |
| pH | **0.296** | 0.138 | 0.138 | 0.196 | 0.357 | 0.317 |
| POR | 0.034 | 0.079 | 0.136 | 0.036 | 0.016 | 0.158 |

Estimates in bold are deemed significantly different from zero (lower CI 95 % > 0.1). H: plant height; S: number of stems; RGR: relative growth rate; AGBM: aboveground biomass; BGBM: belowground biomass; LDMC: leaf dry matter content; Root:Shoot: ratio root to shoot; AG-C:N: carbon to nitrogen ratio of aboveground biomass; BG-C:N: carbon to nitrogen ratio of belowground biomass; AG-N: total aboveground N accumulation; pH: soil pH; POR: root porosity.
