# Supplementary material for: Trait expression and signatures of adaptation in response to nitrogen addition in the common wetland plant Juncus effusus
Source: PLoS One. 2019 Jan 4;14(1):e0209886. doi: 10.1371/journal.pone.0209886 (PMC6319709; doi:10.1371/journal.pone.0209886)
Supplement: S3 Table — (DOCX) [file pone.0209886.s004.docx]

| Functional traits | PC1 | | Treatment | | PC1 × treatment | | PC2 | | Treatment | | PC2 × treatment | |
| --- | --- | --- | --- | --- | --- | --- | --- | --- | --- | --- | --- | --- |
|  | df | F | df | F | df | F | df | F | df | F | df | F |
| H | 1.16 | 0.5 | 2.692 | **108.3***** | 2.679 | 0.6 | 1.22 | 0.6 | 2.698 | **91.9***** | 2.698 | 0.5 |
| S | 1.17 | **7.0*** | 2.699 | **181.5***** | 2.684 | 0.8 | 1.21 | 0.8 | 2.703 | **153.5***** | 2.701 | 0.4 |
| RGR | 1.19 | 0.3 | 2.702 | **151.6***** | 2.623 | 2.1 | 1.38 | 1.8 | 2.700 | **120.1***** | 2.655 | 3.0 |
| AGBM | 1.18 | 3.55 | 2.704 | **405.3***** | 2.689 | **3.1*** | 1.30 | 0.1 | 2.709 | **351.1***** | 2.709 | 0.4 |
| BGBM | 1.18 | **4.5*** | 2.699 | **41.0***** | 2.683 | **5.4**** | 1.12 | 1.0 | 2.701 | **37.9***** | 2.688 | 0.6 |
| LDMC | 1.16 | **5.8*** | 2.706 | **17.9***** | 2.692 | 1.6 | 1.33 | 0.0 | 2.710 | **16.3***** | 2.692 | 0.2 |
| Root:Shoot | 1.16 | 0.0 | 2.675 | **336.0***** | 2.665 | **20.3***** | 1.15 | 0.0 | 2.688 | **264.7***** | 2.695 | 1.4 |
| AG-C:N | 1.15 | **8.7*** | 2.360 | **103.3***** | 2.342 | 1.8 | 1.26 | 1.1 | 2.364 | **82.3***** | 2.372 | 0.9 |
| BG-C:N | 1.18 | **8.6**** | 2.367 | **228.6***** | 2.353 | **5.7**** | 1.31 | 1.1 | 2.371 | **184.7***** | 2.375 | 1.2 |
| AG-N | 1.17 | 0.0 | 2.394 | **389.4***** | 2.378 | 1.2 | 1.29 | 0.2 | 2.401 | **333.7***** | 2.397 | 1.0 |
| pH | 1.44 | 0.2 | 2.441 | **6.1**** | 2.441 | 0.3 | 1.13 | 0.0 | 2.442 | **5.4**** | 2.440 | 1.1 |
| POR | 1.74 | 1.5 | 2.728 | 0.7 | 2.692 | **3.4*** | 1.14 | 0.6 | 2.726 | 0.9 | 2.686 | 0.7 |

**S3 Table. Effects of soil environment of the source location (measured as the first and second axis of a principal component analysis of all soil parameters, PC1 and PC2) and experimental nitrogen addition and their interaction on mean quantitative trait expression in *Juncus effusus*.**

Depicted are F values and significances in bold (*P<0.05, **P<0.01, ***P<0.001) based on mixed effect models and respective analyses of variance with Satterthwaite approximation for degrees of freedom. For trait explanations see Table S2.
