# Supplementary material for: Trait expression and signatures of adaptation in response to nitrogen addition in the common wetland plant Juncus effusus
Source: PLoS One. 2019 Jan 4;14(1):e0209886. doi: 10.1371/journal.pone.0209886 (PMC6319709; doi:10.1371/journal.pone.0209886)
Supplement: S4 Table — (DOCX) [file pone.0209886.s005.docx]

**S4 Table. Effects of soil environment of the source location (measured as the first axis of a principal component analysis of all soil parameters, PC1) and lineage membership and their interaction on mean quantitative trait expression in *Juncus effusus*.**

| N supply | T0 | | | | | | T70 | | | | | | T150 | | | | | |
| --- | --- | --- | --- | --- | --- | --- | --- | --- | --- | --- | --- | --- | --- | --- | --- | --- | --- | --- |
| Functional traits | PC1 | | Lineage | | PC1×  lineage | | PC1 | | Lineage | | PC1×  lineage | | PC1 | | Lineage | | PC1×  lineage | |
|  | df | F | df | F | df | F | df | F | df | F | df | F | df | F | df | F | df | F |
| H | 1.14 | 0.1 | 1.14 | **6.5*** | 1.14 | **5.9*** | 1.8 | 0.2 | 1.7 | **40.4***** | 1.8 | 0.5 | 1.11 | 0 | 1.11 | **10.8**** | 1.11 | 0.2 |
| S | 1.14 | 2.2 | 1.15 | 2.6 | 1.15 | 0.1 | 1.7 | 1.9 | 1.7 | **8.0*** | 1.7 | 1.8 | 1.11 | 3.7 | 1.11 | **20.9***** | 1.11 | 0.8 |
| RGR | 1.14 | 1.2 | 1.14 | 0.1 | 1.14 | 1.1 | 1.7 | 0.0 | 1.7 | 2.4 | 1.7 | 1.8 | 1.11 | 2.1 | 1.11 | 0.6 | 1.11 | **6.9*** |
| AGBM | 1.14 | 0.7 | 1.15 | 0.1 | 1.14 | 1.1 | 1.7 | 0.0 | 1.7 | 0.3 | 1.7 | 1.4 | 1.11 | 2.4 | 1.11 | 1.2 | 1.11 | 0.5 |
| BGBM | 1.15 | 0.0 | 1.15 | 2.2 | 1.15 | 1.7 | 1.7 | 0.4 | 1.7 | 0.0 | 1.7 | 0.4 | 1.11 | **5.8*** | 1.11 | 4.4 | 1.11 | 0.2 |
| LDMC | 1.14 | 3.6 | 1.14 | 0.3 | 1.14 | 0.0 | 1.7 | 0.0 | 1.7 | 0.0 | 1.7 | 1.7 | 1.11 | 4.1^b^ | 1.11 | **4.9*** | 1.11 | 0.1 |
| Root:Shoot | 1.13 | **5.6* ^a^** | 1.14 | **20.1***** | 1.13 | 0.0 | 1.7 | 2.6 | 1.7 | 0.0 | 1.7 | 0 | 1.11 | 1.5 | 1.11 | 1.4 | 1.11 | 0.1 |
| AG-C:N | 1.15 | **14.3** ^a^** | 2.17 | **5.9* ^a^** | 2.16 | **5.9* ^a^** | 1.7 | **15.9**** | 1.7 | 0.4 | 1.7 | 0 | 1.11 | **7.1*** | 1.11 | 0.6 | 1.11 | 0.1 |
| BG-C:N | 1.15 | 0.1 | 1.15 | 0.1 | 1.15 | 1.6 | 1.7 | **13.4**** | 1.7 | 0.1 | 1.7 | 0.3 | 1.11 | 2.4 | 1.11 | 0.1 | 1.11 | 0.0 |
| AG-N | 1.15 | 0.0 | 1.15 | 0.4 | 1.15 | 0.7 | 1.7 | 3.7 | 1.7 | 1.0 | 1.7 | **6.3*** | 1.11 | 0 | 1.11 | 0.0 | 1.11 | 0.2 |
| pH | 1.15 | 1.0 | 1.16 | 0.1 | 1.15 | 2.6 | 1.40 | 0.2 | 1.40 | 1.6 | 1.4 | **6.0*** | 1.11 | 0.1 | 1.11 | 2.1 | 1.11 | 0.2 |
| POR | 1.86 | 0.3 | 1.86 | **4.2* ^a^** | 1.86 | 0.0 | 1.7 | 2.1 | 1.7 | 0.8 | 1.7 | 0.8 | 1.56 | 0.3 | 1.56 | 0.7 | 1.56 | 1.2 |

Depicted are F values and significances in bold (*P<0.05, **P<0.01, ***P<0.001) based on linear models and respective analyses of variances. For trait explanations see Table S2. ^a^ – Not significant when using seed mass as covariate in the model. ^b^ – Becomes significant when using seed mass as covariate.
