# Supplementary material for: Trait expression and signatures of adaptation in response to nitrogen addition in the common wetland plant Juncus effusus
Source: PLoS One. 2019 Jan 4;14(1):e0209886. doi: 10.1371/journal.pone.0209886 (PMC6319709; doi:10.1371/journal.pone.0209886)
Supplement: S5 Table — (DOCX) [file pone.0209886.s006.docx]

**S5 Table. Effects of soil environment of the source location (measured as the second axis of a principal component analysis of all soil parameters, PC2) and lineage membership and their interaction on mean quantitative trait expression in *Juncus effusus*.**

| N supply | T0 | | | | | | T70 | | | | | | T150 | | | | | |
| --- | --- | --- | --- | --- | --- | --- | --- | --- | --- | --- | --- | --- | --- | --- | --- | --- | --- | --- |
| Functional traits | PC2 | | Lineage | | PC2×  lineage | | PC2 | | Lineage | | PC2×  lineage | | PC2 | | Lineage | | PC2×  lineage | |
|  | df | F | df | F | df | F | df | F | df | F | df | F | df | F | df | F | df | F |
| H | 1.15 | 0.2 | 1.15 | 2.2 | 1.15 | 2.4 | 1.7 | 0.6 | 1.7 | 0.2 | 1.7 | 0.7 | 1.11 | 3.9^b^ | 1.11 | 2.5 | 1.11 | **10.9**** |
| S | 1.16 | 0.5 | 1.15 | 1.4 | 1.16 | 0.0 | 1.44 | 0.9 | 1.43 | 2.7 | 1.44 | 2.3 | 1.11 | **13.7**** | 1.11 | **21.5***** | 1.11 | 1.8 |
| RGR | 1.16 | 0.0 | 1.15 | 0.3 | 1.16 | 0.9 | 1.44 | 1.2 | 1.44 | 0.1 | 1.44 | 0.1 | 1.11 | 0.7 | 1.11 | 0.0 | 1.11 | 0.3 |
| AGBM | 1.15 | 1.6 | 1.15 | 0.1 | 1.15 | 0.1 | 1.7 | 0.0 | 1.7 | 0.6 | 1.7 | 1.1 | 1.11 | 0.5 | 1.11 | 4.3 | 1.11 | 4.4 |
| BGBM | 1.16 | 0.6 | 1.15 | 1.9 | 1.16 | 0.03 | 1.7 | 0.1 | 1.7 | 0.5 | 1.7 | 1.1 | 1.11 | 3.5 | 1.11 | **5.7* ^a^** | 1.11 | 3.1 |
| LDMC | 1.15 | 0.4 | 1.15 | 0.2 | 1.15 | 0.2 | 1.7 | 0.1 | 1.7 | 0.6 | 1.7 | 0.9 | 1.11 | 0.4 | 1.11 | 2.4 | 1.11 | 0.3 |
| Root:Shoot | 1.16 | 1.0 | 1.15 | **14.1**** | 1.16 | 0.0 | 1.7 | 1.2 | 1.7 | 0.2 | 1.7 | 1.0 | 1.11 | **5.1*** | 1.11 | 0.1 | 1.11 | 0.5 |
| AG-C:N | 1.17 | 0.1 | 1.15 | 1.9 | 1.17 | 0.1 | 1.7 | **6.0*^a^** | 1.7 | 0.7 | 1.7 | 0.0 | 1.11 | 2.0 | 1.11 | 0.0 | 1.11 | 0.0 |
| BG-C:N | 1.17 | 0.2 | 1.15 | 0.2 | 1.17 | 0.1 | 1.7 | **8.6*^a^** | 1.7 | 2.1 | 1.7 | 0.6 | 1.11 | 3.4 | 1.11 | 1.1 | 1.11 | 0.8 |
| AG-N | 1.16 | 0.3 | 1.15 | 0.4 | 1.16 | 0.0 | 1.7 | 1.4 | 1.7 | 1.6 | 1.7 | 2.1 | 1.11 | 0.0 | 1.11 | 4.2 | 1.11 | **10.9**** |
| pH | 1.20 | 1.6 | 1.16 | 0.1 | 1.20 | 0.2 | 1.42 | 0.0 | 1.42 | 0.4 | 1.42 | 0.3 | 1.11 | 0.0 | 1.11 | **5.8* ^a^** | 1.11 | 3.0 |
| POR | 1.83 | 0.2 | 1.85 | **4.3* ^a^** | 0.83 | 0.1 | 1.43 | 1.7 | 1.43 | **4.6* ^a^** | 1.43 | **7.3**** | 1.11 | 0.0 | 1.11 | 0.4 | 1.11 | 0.0 |

Depicted are F values and significances in bold (*P<0.05, **P<0.01, ***P<0.001) based on linear models and respective analyses of variances. For trait explanations see Table S2. ^a^ – Not significant when using seed mass as covariate in the model. ^b^ – Becomes significant when using seed mass as covariate.
