# Supplementary material for: Trait expression and signatures of adaptation in response to nitrogen addition in the common wetland plant Juncus effusus
Source: PLoS One. 2019 Jan 4;14(1):e0209886. doi: 10.1371/journal.pone.0209886 (PMC6319709; doi:10.1371/journal.pone.0209886)
Supplement: S6 Table — (DOCX) [file pone.0209886.s007.docx]

**S6 Table. Multiple matrix regression with randomization analysis (MMRR) explaining pairwise trait differentiation between populations (*Q*^ij^_ST_) jointly by (A) pairwise soil environmental distance and** **(B) pairwise neutral genetic differentiation (*F^ij^*_ST_) for the subset of 12 *Juncus effusus* populations across treatment conditions and for each treatment T0 (N = 22), T70 (N = 12) and T150 (N = 16) separately.**

|  | Overall | |  | T0 | |  | T70 | |  | T150 | |
| --- | --- | --- | --- | --- | --- | --- | --- | --- | --- | --- | --- |
| Functional traits | (A) | (B) |  | (A) | (B) |  | (A) | (B) |  | (A) | (B) |
| H | 0.015 | **0.249**** |  | **0.095*** | 0.032 |  | -0.034 | **0.252**** |  | -0.065 | **0.115*** |
| S | -0.005 | **0.209**** |  | -0.051 | 0.007 |  | -0.005 | **0.210**** |  | -0.002 | **0.504**** |
| RGR | -0.024 | 0.016 |  | -0.011 | -0.020 |  | 0.001 | -0.009 |  | -0.041 | 0.021 |
| AGBM | 0.025 | -0.056 |  | 0.038 | -0.029 |  | 0.011 | -0.059 |  | 0.036 | -0.061 |
| BGBM | 0.026 | 0.007 |  | 0.001 | -0.023 |  | 0.036 | -0.039 |  | 0.066 | -0.033 |
| LDMC | 0.020 | -0.102 |  | 0.043 | -0.042 |  | -0.017 | -0.054 |  | 0.042 | -0.008 |
| Root:Shoot | 0.000 | 0.015 |  | -0.008 | **0.054**** |  | 0.016 | 0.004 |  | 0.001 | 0.003 |
| AG-C:N | **0.077*** | 0.075 |  | -0.039 | 0.031 |  | **0.238**** | -0.006 |  | **0.153**** | -0.008 |
| BG-C:N | 0.050 | 0.034 |  | -0.011 | 0.014 |  | **0.212**** | 0.012 |  | 0.104 | 0.034 |
| AG-N | 0.000 | 0.002 |  | 0.000 | -0.006 |  | -0.007 | -0.003 |  | -0.004 | -0.006 |
| pH | 0.001 | 0.002 |  | -0.001 | -0.004 |  | -0.001 | 0.008 |  | -0.006 | 0.008 |
| POR | -0.011 | -0.010 |  | -0.005 | 0.019 |  | 0.093 | 0.075 |  | -0.032 | -0.027 |

Values show regression coefficients (r) and bold estimates indicate significant departures from zero (*P<0.05, **P<0.01, ***P<0.001). For trait explanations see Table S2.
