# Supplementary material for: Trait expression and signatures of adaptation in response to nitrogen addition in the common wetland plant Juncus effusus
Source: PLoS One. 2019 Jan 4;14(1):e0209886. doi: 10.1371/journal.pone.0209886 (PMC6319709; doi:10.1371/journal.pone.0209886)
Supplement: S7 Table — (DOCX) [file pone.0209886.s008.docx]

**S7 Table. Multiple matrix regression with randomization analysis (MMRR) explaining pairwise trait differentiation between populations (*Q*^ij^_ST_) jointly by (A) pairwise soil environmental distance and (B) pairwise neutral genetic differentiation (*F^ij^*_ST_) for the lineages Eff1 and Eff2 and treatment conditions (T0, T70 and T150) separately.**

|  | T0 | | | |  | T70 | | | |  | T150 | | | |
| --- | --- | --- | --- | --- | --- | --- | --- | --- | --- | --- | --- | --- | --- | --- |
| Functional traits | Eff1 | | Eff2 | |  | Eff1 | | Eff2 | |  | Eff1 | | Eff2 | |
|  | (A) | (B) | (A) | (B) |  | (A) | (B) | (A) | (B) |  | (A) | (B) | (A) | (B) |
| H | -0.036 | **0.293**** | -0.061 | **0.106*** |  | 0.148 | -0.116 | -0.035 | 0.071 |  | 0.114 | -0.135 | -0,034 | 0,157 |
| S | -0.160 | 0.087 | 0.012 | 0.030 |  | -0.128 | 0.391 | -0.022 | 0.033 |  | 0.108 | 0.135 | -0,018 | 0,186 |
| RGR | **-0.209*** | 0.085 | 0.002 | 0.028 |  | -0.072 | 0.059 | -0.002 | 0.009 |  | 0.014 | -0.078 | -0,032 | 0,122 |
| AGBM | -0.120 | **0.222*** | -0.018 | -0.090 |  | 0.092 | -0.158 | 0.083 | **-0.223*** |  | 0.222 | -0.025 | 0,125 | **-0,334*** |
| BGBM | -0.023 | 0.033 | 0.018 | -0.086 |  | -0.105 | 0.043 | 0.075 | **-0.245*** |  | 0.160 | -0.002 | 0,103 | **-0,271*** |
| LDMC | -0.054 | 0.110 | -0.034 | -0.041 |  | 0.208 | -0.243 | 0.108 | -0.273 |  | **0.221**** | -0.041 | -0,018 | -0,096 |
| Root:Shoot | -0.021 | 0.023 | 0.011 | -0.003 |  | -0.050 | 0.072 | 0.004 | 0.010 |  | 0.023 | 0.005 | 0,000 | 0,031 |
| AG-C:N | -0.034 | 0.025 | -0.050 | 0.030 |  | -0.090 | 0.293 | **0.271*** | -0.121 |  | **0.296***** | 0.051 | 0,033 | 0,027 |
| BG-C:N | -0.028 | 0.044 | -0.017 | 0.052 |  | -0.166 | 0.422 | 0.365 | -0.158 |  | 0.179 | 0.028 | 0,239 | 0,050 |
| AG-N | 0.002 | -0.014 | -0.003 | 0.001 |  | 0.007 | -0.018 | 0.018 | -0.005 |  | 0.003 | -0.005 | 0,003 | -0,013 |
| pH | -0.006 | 0.005 | 0.007 | -0.016 |  | 0.018 | 0.005 | -0.023 | **0.024*** |  | -0.022 | 0.011 | -0,013 | 0,021 |
| POR | -0.021 | -0.053 | 0.005 | 0.000 |  | -0.067 | 0.305 | -0.237 | 0.236 |  | -0.036 | 0.005 | -0,045 | -0,062 |

Values show regression coefficients (r) and bold estimates indicate significant departures from zero (*P<0.05, **P<0.01, ***P<0.001). For trait explanations see Table S2.
