# Supplementary material for: Trait expression and signatures of adaptation in response to nitrogen addition in the common wetland plant Juncus effusus
Source: PLoS One. 2019 Jan 4;14(1):e0209886. doi: 10.1371/journal.pone.0209886 (PMC6319709; doi:10.1371/journal.pone.0209886)
Supplement: S1 Fig — Circles represent individuals grouped in three distinct clusters indicated by colors (red: Eff1, blue: Eff2, green: Eff3). (DOCX) [file pone.0209886.s009.docx]

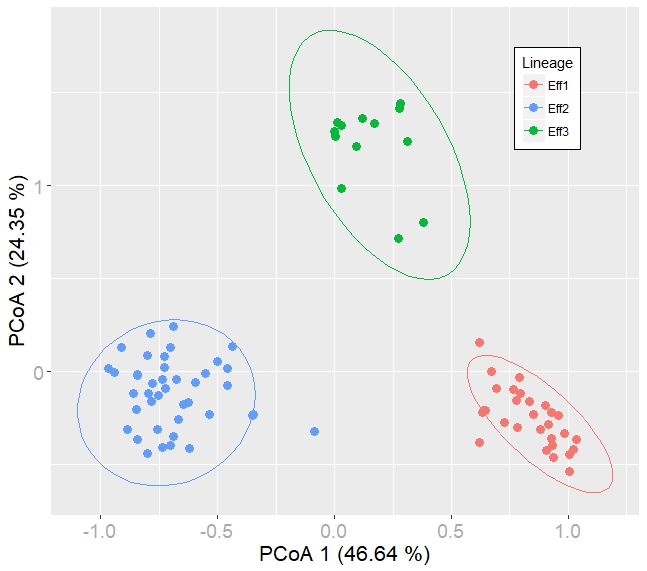


S1 Fig. Principal coordinate analysis (PCoA) plot based on pairwise genotypic distances among individuals. Circles represent individuals grouped in three distinct clusters indicated by colors (red: Eff1, blue: Eff2, green: Eff3).
