# Supplementary material for: Trait expression and signatures of adaptation in response to nitrogen addition in the common wetland plant Juncus effusus
Source: PLoS One. 2019 Jan 4;14(1):e0209886. doi: 10.1371/journal.pone.0209886 (PMC6319709; doi:10.1371/journal.pone.0209886)
Supplement: S2 Fig — Grey arrows indicate loadings of each soil variable on the two axes (particle size distribution: clay-, silt- and sand content, coarse fragments, pH (CaCl2), organic carbon (OC), potassium- (K), phosphorus- (P), carbonate- (CACO3) and total nitrogen (N) content and cation exchange capacity (CEC)), while colored symbols represent populations and their underlying genotype (red: Eff1, blue: Eff2, green: Eff3) in the environmental space. (DOCX) [file pone.0209886.s010.docx]

**
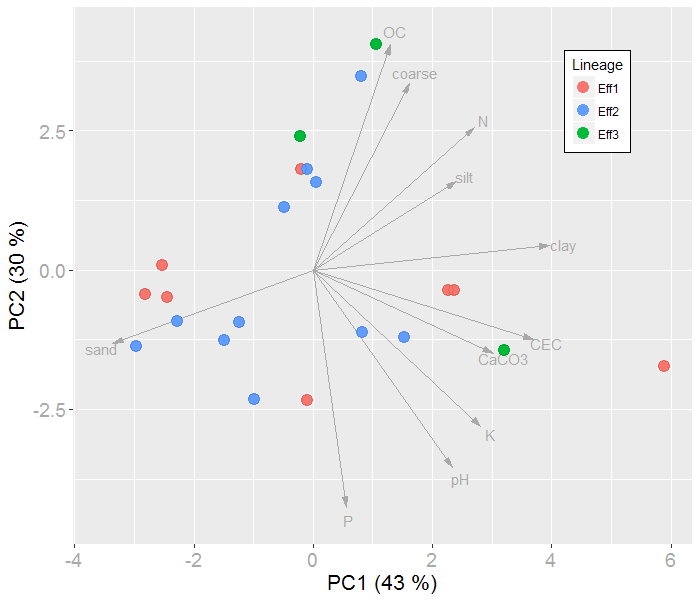
**

**S2 Fig. The Principal component analysis (PCA) of 11 soil variables for 22 *Juncus effusus* populations.** Grey arrows indicate loadings of each soil variable on the two axes (particle size distribution: clay-, silt- and sand content, coarse fragments, pH (CaCl_2_), organic carbon (OC), potassium- (K), phosphorus- (P), carbonate- (CACO_3_) and total nitrogen (N) content and cation exchange capacity (CEC)), while colored symbols represent populations and their underlying genotype (red: Eff1, blue: Eff2, green: Eff3) in the environmental space.
