# Supplementary material for: Trait expression and signatures of adaptation in response to nitrogen addition in the common wetland plant Juncus effusus
Source: PLoS One. 2019 Jan 4;14(1):e0209886. doi: 10.1371/journal.pone.0209886 (PMC6319709; doi:10.1371/journal.pone.0209886)
Supplement: S3 Fig — (DOCX) [file pone.0209886.s011.docx]

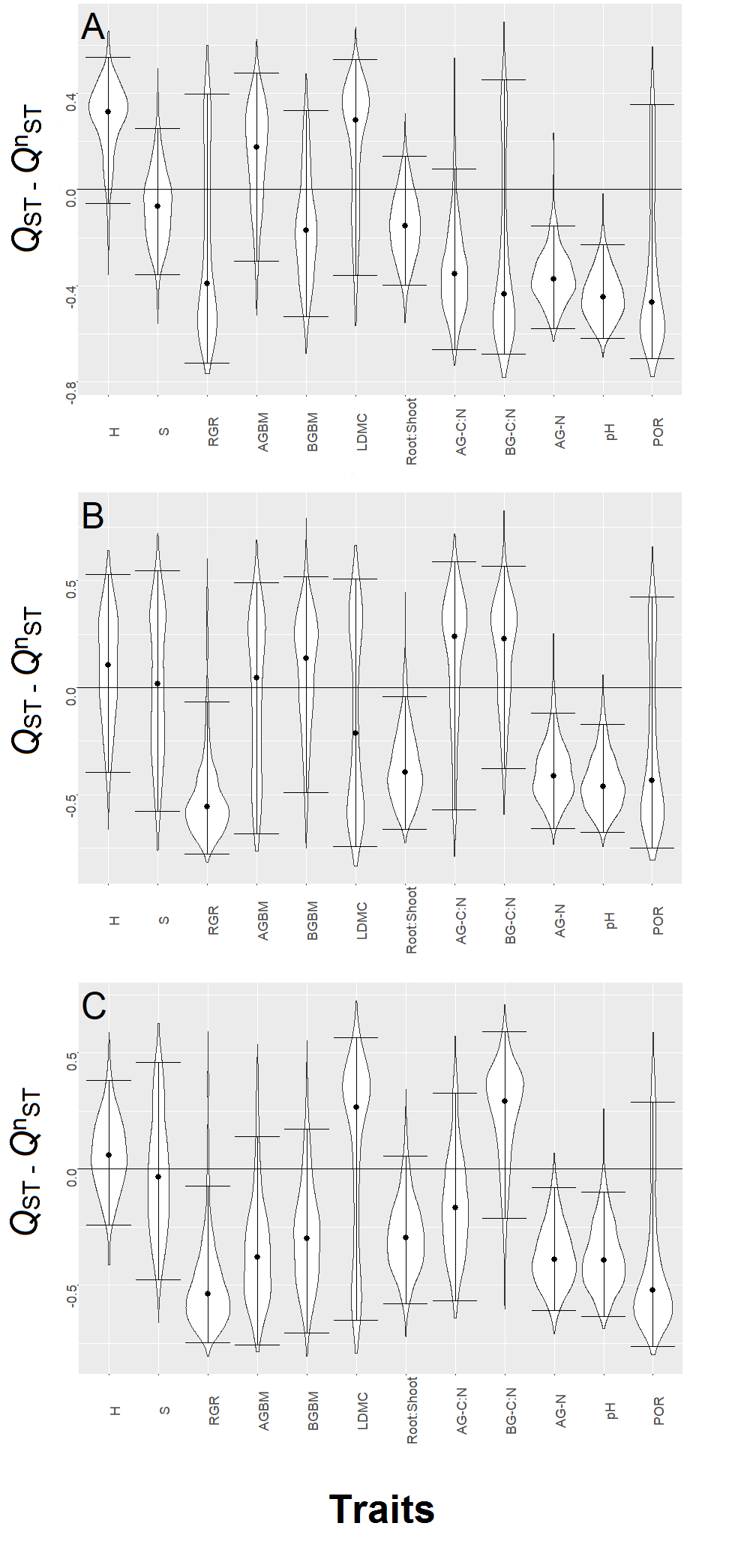
**S3 Fig. Violin plot shows the comparison of quantitative genetic divergence (*Q*_ST_) and with the expected distribution under neutrality (*Q*^n^_ST_) for each treatment separately (A: T0, N = 22; B: T70, N = 12 and C: T150, N = 16).** For trait explanations see Table S2.
