# Supplementary material for: Trait expression and signatures of adaptation in response to nitrogen addition in the common wetland plant Juncus effusus
Source: PLoS One. 2019 Jan 4;14(1):e0209886. doi: 10.1371/journal.pone.0209886 (PMC6319709; doi:10.1371/journal.pone.0209886)
Supplement: S4 Fig — (DOCX) [file pone.0209886.s012.docx]

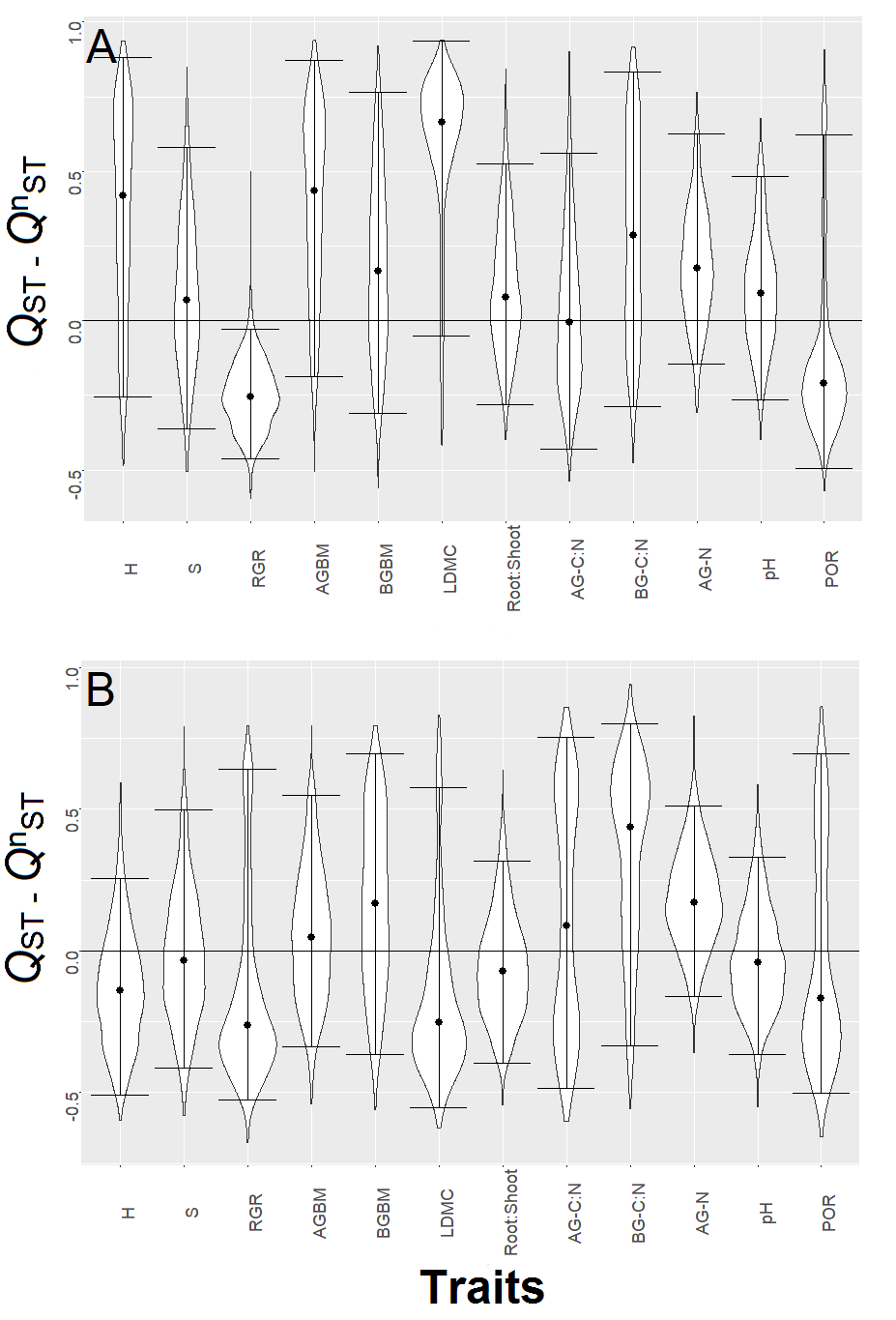
**S4 Fig. Violin plot shows the comparison of quantitative genetic divergence (*Q*_ST_) and with the expected distribution under neutrality (*Q*^n^_ST_) within detected lineages Eff1 (A, N = 8) and Eff2 (B, N = 11).** For trait explanations see Table S2.
